# Reluctancy towards Help-Seeking for Mental Health Concerns at Secondary School among Students in the COMPASS Study

**DOI:** 10.3390/ijerph17197128

**Published:** 2020-09-29

**Authors:** Natalie Doan, Karen A. Patte, Mark A. Ferro, Scott T. Leatherdale

**Affiliations:** 1 School of Public Health and Health Systems, University of Waterloo, Waterloo, ON N2L 3G1, Canada; mark.ferro@uwaterloo.ca (M.A.F.); sleatherdale@uwaterloo.ca (S.T.L.); 2 Department of Health Sciences, Brock University, St. Catharines, ON L2S 3A1, Canada; kpatte@brocku.ca

**Keywords:** school mental health, school psychology, help-seeking, mental health, adolescence, youth, students

## Abstract

Youth populations represent a key population for addressing mental health, yet many youths express reluctance towards help seeking. Considering the volume of time that almost all youth spend at school during the school year, it is important to assess the role of the school environment in relation to students’ attitudes toward help-seeking. Data from 47,290 grade 9 to 12 students and 116 Canadian secondary schools that participated in the 2018-19 wave of the COMPASS (Cannabis, Obesity, Mental health, Physical activity, Alcohol, Smoking, Sedentary behaviour) study were analyzed using GEE models to assess the student and school characteristics associated with attitudes regarding seeking help for mental health concerns from an adult at school. Overall, 58% of students reported being reluctant to seek help for mental health concerns at school. Students who reported lower self-rated mental health (aOR = 1.76, 95% CI = 1.65, 1.87), emotion regulation (aOR = 1.08, 95% CI = 1.07, 1.09), family support (aOR = 2.31, 95% CI = 2.16, 2.47), peer support (aOR = 1.20, 95% CI = 1.13, 1.31), and school connectedness (aOR = 0.93, 95% CI = 0.92, 0.93) scores were more likely to be reluctant towards help-seeking at school than students with more favourable scores on these variables. Students with higher flourishing scores were less likely than students who were languishing to report reluctance to help-seeking at school (aOR = 0.96, 95% CI = 0.96, 0.97). Students attending schools in areas with lower population densities and median household incomes between $50,000–75,000 were less likely to be reluctant to help-seeking relative to students attending schools in areas with higher density (aOR = 0.85, 95% CI = 0.79, 0.93) and median household incomes (aOR = 1.20, 95% CI = 1.13, 1.31), respectively. The availability of school mental health services and specialists were not associated with student help-seeking reluctance. High levels of resistance towards help-seeking among youth remain a significant barrier, particularly among youth at highest risk (i.e., with lower support and poorer mental health). The student and school characteristics identified in the current study can help inform strategies to promote greater acceptance of help seeking among students in schools.

## 1. Introduction

Globally, mental illnesses account for approximately one-third of the burden of illnesses among young people [1]. Given that approximately 70% of future adult mental illnesses emerge during adolescence [2], prevention and early intervention efforts targeting youth populations are essential. Despite the recognized importance of addressing youth mental health, young people’s reluctancy towards help-seeking continues to impede prevention and early detection efforts [3]. A systematic review by Gulliver and colleagues concluded that stigma, embarrassment, poor mental health literacy, and a preference for self-reliance are top barriers towards help-seeking among youth [4]. Addressing the barriers associated with help-seeking is critical to prevent the escalating severity and chronicity of symptoms because untreated mental health problems and illnesses that are associated with poor vocational achievements, problematic interpersonal and family relationships, reduced life expectancies due to related medical conditions (e.g., diabetes, coronary heart disease), and suicide [5,6,7,8].

According to the Determinants of Health Model (2016), it is important to consider multiple levels of influence when attempting to understand the health and health behaviours of youth [9]. This model presents a framework that can be used to conceptualize the interrelationships between individual indicators of health and collective well-being and encourages us to think about young people’s health within the context of broader networks [9]. Specifically, this model highlights the importance of considering factors located in multiple levels that may influence the health and well-being of youth, including indicators at the individual, family community, and systems level [9]. With respect to understanding youth’s attitudes towards help-seeking for mental health concerns, indicators of psychological health and social well-being are considered important determinants of health at the individual level [9]. The general consensus in the literature is that youth who experience poor mental health are less likely to seek help than youth who report good mental health [3,10]. In addition, indicators of social well-being, such as a sense of connectedness to friends and family, is believed to foster a healthy sense of belonging, self-efficacy, self-regard, and confidence [11] that could be associated with positive help-seeking behaviours. However, the research on social support and help-seeking remains unclear, with some research suggesting that adolescents with more close friends are less likely to seek help from formal supports [12]; while other research suggests that social support is positively associated with an acceptance towards help-seeking [11]. Lastly, the sociodemographic characteristics of individuals, such as sex, gender, race, and ethnicity, are strongly associated with attitudes towards help-seeking [10,13]. Thus, the psychological, social, and sociodemographic characteristics of youth must be considered when examining the help-seeking attitudes of youth.

The Determinants of Health Model also recognizes the profound influence of broader contexts, such as the school setting, on the health and well-being of youth, particularly among high-risk and/or marginalized populations [9,14]. Considering that schools have unparalleled contact with youth, schools are an opportune setting to reach those who have not been previously identified and/or treated for a mental health problem [15,16]. Since many schools offer free mental health resources on-site, the practical barriers associated with accessing community-based treatments are often mitigated (e.g., time, travel, cost) [17,18]. Additionally, schools are ideal contexts for both universal mental health promotion efforts for the full student body and targeted prevention efforts for students placed at-risk [19]. However, it is not clear which direction schools can take to improve an acceptance towards help-seeking among their students based on their student populations and school setting [20]. Based on the theoretical frameworks of the Determinants of Health Model, research examining links between the school environment and help-seeking attitudes is needed to better understand how schools can better support the mental health needs of youth [21], especially by using a large population study with linked student- and school-level data that enable the examination of help-seeking attitudes. Therefore, the primary objective of this project was to use hierarchical data from the COMPASS host study to simultaneously explore the associations between school characteristics and students’ attitudes towards help-seeking for mental health concerns at school. By investigating these associations, researchers can better understand how school stakeholders can take action to support a positive attitude towards help-seeking for mental health concerns at school.

## 2. Materials and Methods

### 2.1. Study Design

Linked student- and school-level data from wave 7 of the COMPASS study (Year 7; Y7 (2018,2019)) were examined. The COMPASS study is a 9-year prospective cohort study (2012–2021) collecting data from a convenience sample of schools and students attending those schools annually to assess how changes in the school environment are associated with changes in youth health behaviours over time [22]. Student-level data collected were linked with school-level data collected from a convenience sample of schools in Quebec, Ontario, Alberta, and British Columbia. A purposeful sampling procedure was utilized to recruit schools that permit the use of an active-information and passive consent protocols [23]. Full school samples were collected, with all students attending participating schools considered eligible to participate in the study if (1) their parents/guardian did not inform the recruitment coordinator that they did not want their child to participate and (2) the student agreed to participate. All students were informed that they could withdraw their participation at any time [23]. Student-level data were collected using paper-and-pencil questionnaires (Cq) completed during one classroom period. School-level data were collected at the same time as the student data collection using an online survey (the School Policies and Practice Questionnaire (SPP)) completed by the school contact(s) that is most knowledgeable about their school’s health programs and policies offered [22]. For this study, we are only using Y7, data collected from 116 schools that had also participated in Y6 (2017–2018) as the Y6 data were used to calculate on of our school-level predictor variables. As such, the final analytic sample for this paper included 47,290 students attending 116 schools. All procedures have been approved by the University of Waterloo Office of Research Ethics (ORE 30118), and all required participating school board and/or school ethics committees. Additional details of the COMPASS study are available in print [22] and online (https://uwaterloo.ca/compass-system/).

Availability of Data and Materials: COMPASS data are available for researchers upon request through completion and approval of the COMPASS data usage application available online (https://uwaterloo.ca/compass-system/information-researchers).

### 2.2. Measures

Please refer to Appendix A for a copy of the COMPASS study survey measures that were used in the present study.

#### 2.2.1. Mental Health Help-Seeking Attitudes

Using measures consistent with other youth population studies [24,25,26,27], students were asked to report their attitudes towards help-seeking within their school environment. The student questionnaire item asked students: “If you had concerns regarding your mental health, are there any reasons why you would not talk to an adult at school (e.g., a school social worker, child and youth worker, counsellor, psychologist, nurse, teacher, or other staff person)?” (Mark all that apply). Response options included: “I would have no problems talking to an adult at school about my mental health”, “Worried about what others would think of me (e.g., I’d be too embarrassed)”, “Lack of trust in these people—word would get out”, “Prefer to handle problems myself”, “Do not think these people would be able to help”, “Would not know who to approach”, and “There is no one I feel comfortable talking to.” A binary variable was derived by categorizing students as “reluctant” if they endorsed one or more deterrent towards help-seeking, or ‘not reluctant’ if students did not select any deterrents to help-seeking at school.

#### 2.2.2. School-Level Variables

To gauge the extent to which mental health is a school priority, school contacts were asked on the SPP to: “Please rank these school/health-related issues in terms of importance to your school: (Rank items from 1 to 10 where 1 = highest priority and 10 = lowest priority)”. The issues schools were asked to rank were: tobacco use, alcohol and other drug use, healthy eating, physical activity, bullying/violence, mental health, sexual health, sun safety/tanning beds, obesity/overweight/healthy weight, and sedentary behaviours/screen time. Mental health was considered a “high” priority at a school when it was ranked between 1 and 3 and “low” when it was ranked between 4 and 10.

The availability of mental health professionals at schools was assessed by the SPP item asking school administrators to indicate the availability of child and youth workers, counsellors, social workers, psychologists, mental health nurses, and other mental health professionals at their schools. Mental health professionals that were full-time or regularly scheduled for ≥ 3 times/week or ≥ 16 h/week were considered available. The possible range for the number of mental health professionals available at a school on this scale ranged between 0 and 6, where schools with zero mental health professionals were categorized into the “low/none” category, 1 to 2 into the “medium” category, and 3 to 6 into the “high” availability category.

The availability of mental health services at schools (i.e., on-site individual, group, and family therapy) was assessed by asking administrators on the SPP to indicate the availability of these mental health services at their school (yes, no). This variable was treated as a categorical variable where schools indicating that they had ≤1 of these mental health services were considered ‘low’ and schools with 2–3 of these services were considered “high” in terms of mental health service availability.

General school characteristics were also included in the models. These included indicators representing student enrollment, and school area population density (i.e., urbanicity) and median household income. Student enrollment for the 2018–2019 school year was categorized into three categories: 0 to 500, 501 to 1000, and 1001 to 1500 students. To represent the population density and median household income of the school site, data extracted from the latest census survey were mapped to school postal codes [28]. For the purpose of this study, schools located in rural or small population centres were considered “rural/small urban” and schools located in medium or large population centres were considered “medium/large urban”. Lastly, estimates of school area median household incomes were classified into four categories: $25,000–50,000; $50,000–75,000; $75,000–100,000; and >$100,000.

In order to prevent ecological fallacy [29], Y6 (2017–2018) student data for the 116 participating schools in our sample were used to calculate the school-specific prevalence of students who reported that they had “poor” mental health in the year prior to the data being used in this study, as per the student self-rated mental health variable described below.

#### 2.2.3. Student-Level Variables

As an indicator of self-rated mental health, students were asked “In general, how would you rate your mental health?”. Students were provided with options to indicate “excellent”, “very good”, “good”, “fair”, and “poor” mental health. For the purpose of this study, ratings of excellent, very good, or good were collapsed into one category of generally “good” mental health, whereas ratings of fair or poor were considered “poor”.

Socio-emotional skills were assessed using a modified version of the Difficulties in Emotional Regulation Scale (DERS) [30], that included one item from each of the DERS six subscales (27). Students were asked to indicate their agreement to statements on emotional clarity, emotional awareness, goal-directed behaviours, emotional regulation strategies, impulse control, and non-acceptance of emotional response. A 5-point Likert scale was provided for each item (i.e., almost never = 1, sometimes = 2, almost half the time = 3, most of the time = 4, almost always = 5). By summing the responses to each item, a continuous variable was derived for the analyses where higher scores indicated higher socio-emotional skills. This scale demonstrated acceptable internal consistency among this study’s student sample (α = 0.77).

The presence of self-rated positive psychosocial well-being was assessed using the Flourishing Scale [27]. The items on this scale asked students to indicate their agreement to aspects of psychological and social well-being, such as life satisfaction and meaning, optimism, perceived competence, and relationships using a 5-point Likert scale [27,31]. A continuous sum score variable was derived, with higher scores indicating higher psychological well-being. This scale demonstrated excellent internal consistency among students in this study (α = 0.90).

Two items from the Multidimensional Scale of Perceived Social Support (MSPSS) [32] were used to examined family and peer support. For each of these items, students were asked to indicate the extent to which they agree/disagree that they can talk about their problems with their family/friends on a 5-point Likert scale (i.e., strongly agree, agree, neither agree or disagree, disagree, strongly disagree). Students who endorsed either strongly agree or agree were categorized as agreeing with the statement, either strongly disagree or disagree were categorized as disagreeing with the statements, and students who endorse neither agree nor disagree were considered neutral/ambivalent. Using these items, two separate variables were created to reflect students’ self-perceived family and peer support with three levels of support: “high”, “neutral/ambivalent”, and “low”.

To capture a sense of belonging, satisfaction, and safety at school, six items from the School Connectedness Scale [33] that used a 4-point Likert scale were included in the questionnaire. Students were asked to indicate how strongly they agree or disagree with each item about school connectedness. The scores for each item were summed and recoded into a continuous variable where lower scores indicated that the student feels disconnected from their school community whereas higher scores indicated that the student feels closely connected with their school community. This scale demonstrated good internal consistency among students in this study (α = 0.82).

Bullying victimization was assessed by using a modified version of the bullying measure used in the Ontario Student Drug Use and Health Survey [25,27]. Students were asked if they have been bullied (e.g., physically, verbally, cyber-attacked) by other students in the past 30 days. Students who indicated that they have not been bullied in the past 30 days were classified as “not bullied” and students who selected any of the other options were classified as being “bullied”.

Sociodemographic characteristics were included in models to adjust for their potential confounding effect on the relationships examined. Specifically, all models adjusted for student-reported grade (9, 10, 11, 12), sex (male, female), race/ethnicity (White, Black, Asian, Indigenous (First Nations, Métis, Inuit), Latin American/Hispanic, and Other), and weekly spending/saving money ($0, $1 to $5, $6 to $10, $11 to $20, $21 to $40, $41 to $100, more than $100, I do not know). The race/ethnicity and weekly spending money variables were collapsed into categories of White, Black, Asian, and Other (Indigenous, Latin American/Hispanic, Other, Mixed) and $0, $1 to $20, $21 to $100, more than $100, and I don’t know, respectively.

### 2.3. Analyses

All analyses were conducted using SAS [34]. Descriptive analyses were used to assess the distribution of the explanatory and dependent variables. Generalized linear mixed modelling (GLMM) was used to identify any inherent correlation between students given their school clusters. Generalized equation estimation (GEE) models were used to adjust for the effect of school clustering. In total, four models were tested. Model I tested a null model with none of the explanatory variables entered. Model II tested for the crude relationships between school characteristics and help-seeking. In Model III, the student variables were entered into the model to derived adjusted estimates for the school and student characteristics. Lastly, Model IV tested the presence of an interaction effect between the student and school variables.

## 3. Results

### 3.1. Sample Characteristics

Among participating students, the majority identified as White (66%) and there was an approximately even distribution of students who identified as males (51%) and females (49%). As shown in Table 1, the majority of students reported good mental health (76%), although male students (83%) reported good mental health at a greater frequency than female students (70%). The majority of students reported having high family (58%) and peer support (77%); male students (61%) reported high family support at a greater frequency than female students (55%).

More than half of the students reported reluctance towards help-seeking (58%), with more frequent reluctance among students who identified as female (65%) compared to male (49%). The most commonly reported reasons that students indicated for reluctance towards help-seeking at school included: preferring to handle problems by themselves (34%), worries about what others would think about them (22%), and lacking trust in confiding with the adults at their school (21%).

As shown in Table 2, among the 116 participating schools, half reported having 0–500 students (50%) and there was an approximately equal number of rural/small (56%) and medium/large urban (44%) schools. The majority of school areas had a median household income in the range of $50,000–75,000 (59%). Overall, 84% of schools identified mental health as a high priority in their school, and the majority of schools reported a medium availability level of mental health professionals (63%) and a low level of mental health service availability (69%).

The intraclass correlation estimate (ICC) indicated that the variance between schools needed to be considered in subsequent models (ICC = 2.4%). The ICC suggests that 2.4% of the variance in students reluctancy towards seeking help at their school is a function of the characteristics of the school they attend. Thus, GEE was used to examine the relationship between student characteristics and school factors.

### 3.2. Student and School Characteristics Associated With Reluctancy Towards Help-Seeking

As shown in Table 3, self-rated mental health, socio-emotional skills, and flourishing scores were significantly associated with help-seeking. Students who rated their mental health as poor were more likely to endorse reluctancy towards help-seeking than their peers who reported good mental health (aOR = 1.76, 95% CI = 1.65, 1.87). Everyone unit increase on the emotion regulation scale was associated with greater odds of being reluctant towards help-seeking (aOR = 1.08, 95% CI = 1.07, 1.09). Conversely, everyone unit increase on the Flourishing Scale was associated with a lower odds of being reluctant towards help-seeking (aOR = 0.96, 95% CI = 0.96, 0.97).

Self-reported family support, peer support, school connectedness, and bullying victimization were also significantly associated with help-seeking. When compared to students who reported high family support, students who were neutral/ambivalent regarding their family support were at greater odds of being reluctant towards help-seeking (aOR = 1.74, 95% CI = 1.65, 1.84), and students who reported low family support were at even greater odds of being reluctant towards help-seeking (aOR = 2.31, 95% CI = 2.16, 2.47). Similarly, students who reported neutral/ambivalent (aOR = 1.21, 95% CI = 1.13, 1.31) or low (aOR = 1.20, 95% CI = 1.31, 1.30) peer support had greater odds of reporting reluctancy towards help-seeking. Higher scores on the school connectedness scale were associated with a lower odds of being reluctant towards help-seeking (aOR = 0.93, 95% CI = 0.92, 0.93) and students who were bullied had greater odds of reporting being reluctant towards help-seeking than students who were not bullied (aOR = 1.17, 95% CI = 1.09, 1.26).

Sex, grade, race/ethnicity, spending money, and province were significantly associated with help-seeking among youth in the study. Specifically, the odds of reporting reluctancy towards help-seeking were higher for students who identified as females when compared to males (aOR = 1.70, 95% CI = 1.63, 1.78). Compared to students in Grade 9, Grade 11 students were slightly more likely to be reluctant towards help-seeking (aOR = 1.09, 95% CI = 1.03, 1.15). Students who identified as Black (aOR = 0.87, 95% CI = 0.79, 0.97), Asian (aOR = 0.86, 95% CI = 0.77, 0.96), or Other (aOR = 0.93, 95% CI = 0.99, 0.99) race/ethnicity had a slightly lower odds of endorsing reluctancy towards help-seeking compared to students who identified as White. Lastly, students who were unsure about how much money they had to spend/save each week had lower odds of endorsing reluctancy compared to students who reported $1-20 weekly (aOR = 0.88, 95% CI = 0.88, 0.98).

As shown in Model 4 (Table 3), none of the school-level mental health characteristics were significantly associated with student reluctance toward help-seeking. Additionally, the adjusted model revealed that urbanicity and school area median household income were the only school variables significantly associated with help-seeking. Specifically, students attending schools that were classified as being located in rural or small urban areas had lower odds of endorsing reluctancy towards help-seeking than similar students attending medium or large urban schools (aOR = 0.85, 95% CI = 0.79, 0.93). Similar to the results of the unadjusted model, students attending schools where the school area median household income exceeded $100,000 had higher odds of being reluctant towards help-seeking than similar students attending schools where the school area median household income was between $50,000–75,000 (aOR = 1.20, 95% CI = 1.01, 1.43). Lastly, none of the interaction terms were significantly associated with help-seeking (see Table 3).

## 4. Discussion

### 4.1. Summary of Findings

Given the limited population-based research examining help-seeking attitudes among youth, research examining the student and school factors associated with a reluctancy towards help-seeking is necessary to help inform strategies for mental health promotion and the prevention and early identification of mental health problems and illnesses. This study was the first to examine student and school predictors of students who report being reluctant towards help-seeking at school for mental health concerns in a large sample of Canadian youth. The findings revealed that more than half of students would be reluctant to seek help from an adult at school if they had mental health concerns. Of particular concern, students who had poor mental health, by either the general self-rated item or languishing scores on the Flourishing scale, were at greater odds of being reluctant towards help-seeking at school when compared to students with good mental health, especially females. In addition, students who reported having low social support were at greater odds of being reluctant towards help-seeking at school when compared to students who endorse high levels of social support. Results highlight the need to foster an acceptance towards help-seeking at schools. School and student-level factors associated with reluctancy towards help-seeking in the current study may help inform targeted interventions to improve student willingness to seek help and access school mental health resources.

Results regarding mental health help-seeking attitudes were not surprising as previous research has demonstrated that reluctancy towards help-seeking is frequently endorsed among young people [4]. However, in comparison to studies examining help-seeking in the context of mental disorders (18–34%) [4,35,36,37,38], a higher percentage of students endorsed reluctancy towards help-seeking for mental health concerns in this study. The deterrents most frequently endorsed by youth who were reluctant to seek help suggest that a preference for self-reliance and concerns regarding social disapproval and confidentiality are common reasons why many youths do not feel comfortable seeking help for their mental health at school. These findings are consistent with previous research that indicated that a preference for self-reliance and concerns regarding confidentiality are significant barriers to adolescents seeking help for their mental health concerns in the school setting [4,38,39]. Interventions addressing these potential barriers are important to ensure youth receive appropriate support if they have mental health concerns.

Few general characteristics of the school environment were associated with help-seeking attitudes. The findings revealing the associations between several school characteristics and help-seeking attitudes provides novel findings to add to the body of literature regarding help-seeking attitudes among youth. Students attending rural or small urban schools were at greater odds of being reluctant towards help-seeking at school than students attending medium or large urban schools. This finding is contrary to the literature that demonstrates the high rate of reluctancy towards help-seeking in rural populations [4,40,41,42,43]. Given that this study only examines attitudes towards help-seeking in the school environment, lower rates of reluctance may have been reported by students living in rural areas due to less practical barriers (e.g., distance) associated with accessing mental health resources at school compared to accessing mental health resources in the communities. Although the ICC suggests that it is important to consider the influence of the school environment, the present study did not reveal any significant findings for the associations between any of the school mental health variables and help-seeking attitudes. Various factors may account for this result, including a generally high rate of reluctancy towards help-seeking impacting many students. If this is the case, addressing the deterrents towards help-seeking may help maximize the benefits of offering on-site mental health professionals and services at school. Regardless, it is evident that there is substantial room for improvement that school-based mental health and help-seeking interventions can address.

The present study identifies several student characteristics associated with help-seeking attitudes that should be considered in targeted mental health interventions at school. Students who reported poor mental health, as inferred from self-rated mental health, poor emotion regulation, and low flourishing scores, were at a significantly higher odds of being reluctant towards help-seeking at school. These findings are supported by the body of literature that suggests adolescents with poor mental health are less willing to seek help for their mental health than adolescents who report good mental health [3,10,44]. While not surprising, the findings are concerning because they suggest that students who are in the greatest need of assistance for their mental health may be among the least likely to seek help. Moreover, students who reported they were unable or ambivalent about being able to talk to their family or friends about their problems were at higher odds of being reluctant towards help-seeking compared to students with higher social wellbeing. Previous research suggests social support is associated with positive mental health [11], but the relationship between social support and help-seeking is unclear [3]. The present study results align with the body of research that suggests being integrated into a strong social network has a positive influence not only on mental health but also the help-seeking attitudes of youth [11]. These findings highlight the importance of considering the influence of students’ perception of social support on willingness to seek help for mental health concerns in school-based help-seeking interventions. Although some of the characteristics identified in this study are not necessarily modifiable, the consistently significant findings for the relationships between psychological and social indicators of health with help-seeking attitudes suggest that youth who struggle with their mental health and/or in receiving adequate social support should be considered a priority population in school-based, targeted, help-seeking interventions.

Schools looking to address help-seeking at schools using a universal approach should consider implementing interventions that seek to reduce help-seeking deterrents identified in this study. One possible intervention that has demonstrated to be positively associated with modifying students’ stigmatizing beliefs about mental illnesses in the school setting is contact-based education [45]. Contact-based education operates under the theory that strategies that aim to incorporate education and contact, such as correcting myths and safely sharing personal stories of lived experience with mental illnesses, can reduce public stigma towards individuals with mental illnesses when done properly [46]. Another potential strategy to enhance help-seeking is to provide students with the opportunities to learn socio-emotional techniques that have demonstrated to promote positive help-seeking attitudes among youth, such as coping strategies that support seeking help from friends, family, and professionals [47]. Schools hoping to promote positive help-seeking attitudes and behaviours among their students could consider implementing either of these interventions because of their potential to positively impact students’ beliefs, attitudes, and behaviours for a large number of students [45,48], although understanding their impact would require evaluation.

### 4.2. Strengths and Limitations

The design of this study has many strengths. First, this study used a population survey to examine the help-seeking attitudes of youth attending schools across the country. To date, the large majority of research on help-seeking attitudes among youth populations have been conducted in clinical populations which does not permit examination of help-seeking in the general youth population, especially youth who have not been previously identified as having a mental illness. Additionally, the sample size of schools analyzed in this study is relatively large for research in this field, which allows for greater generalizability and application in the public health context. Lastly, the hierarchical nature of the data analyses accounted for the clustering of student and school characteristics which, to the author’s knowledge, has not been done in the context of help-seeking attitudes among Canadian youth populations.

Despite the strengths of the present study, there are limitations to consider. First, it is important to note that the variable used to examine help-seeking attitudes was posed in a hypothetical manner which necessitates consideration when interpreting the findings as representing students’ current experiences and attitudes towards help-seeking. Second, the complete-case technique employed in this study must be taken into consideration. Only data from students with complete responses to all variables included in the models were included in the analyses, and therefore, the precision of the estimates could have been influenced by response bias. Similarly, the self-report format of the surveys introduces the influence of self-report biases, such as social desirability and recall bias. However, the effect of response and social desirability biases was partly mitigated by the active-information passive-consent procedure of the COMPASS study [23,49] and the measures are consistent with other national population studies and have demonstrated validity and reliability in youth populations [22]. To address the self-report biases introduced using self-report surveys to assess mental health and social well-being, future research could extend this research by interviewing the parents/guardians and youth participant to confirm the findings of the present study. Employing this method may allow researchers to collect relational data and consider the influence of other contextual factors that were not examined in the present study, such as socioeconomic status. Additionally, observational methods can be used to address the limitations associated with survey methods to school-level data collection. Lastly, due to the cross-sectional data analysis design, it is not possible to infer the direction and temporality of the relationships observed. Given the prospective cohort design of the COMPASS study, issues related to temporality and causality can be mitigated in future research by employing longitudinal analyses.

## 5. Conclusions

Fostering positive mental health among youth is of recognized importance, with schools are recognized as key contexts for universal interventions. Similarly, the early identification of mental health problems is critical, to prevent the onset of mental illness and the chronicity and severity of symptoms. However, high levels of resistance towards help-seeking among youth remains a significant barrier to meeting the mental health needs of youth. In corroboration with previous research, the findings from the present study highlight the need for targeted help-seeking interventions at school. By identifying the school and student characteristics associated with help-seeking attitudes, the findings from this study provide promising directions to improve school mental health efforts and future research.

## Figures and Tables

**Table 1:** Demographic and psychosocial characteristics of students participating in Year 6 and 7 (2017–2018, 2018–2019) of the COMPASS Study in Canada (N = 47,290).

Variable	Total N (%)	Males N (%)	Females N (%)
Province			
Ontario (ref.)	26,539 (56%)	13,041 (57%)	13,498 (56%)
Alberta	2955 (6%)	1445 (6%)	150 (6%)
British Columbia	8520 (18%)	4260 (19%)	4260 (17%)
Quebec	9276 (20%)	4249 (18%)	5027 (21%)
Sex			
Males (ref.)	22,995 (49%)	-	-
Females	24,295 (51%)	-	-
Grade			
9 (ref.)	12,684 (26%)	6147 (27%)	6537 (27%)
10	13,484 (29%)	6461 (28%)	7023 (29%)
11	12,804 (27%)	6237 (27%)	6567 (27%)
12	8318 (18%)	4150 (18%)	4168 (17%)
Race/ethnicity			
White (ref.)	31,157 (66%)	15,022 (65%)	16,135 (66%)
Black	1583 (3%)	891 (4%)	692 (3%)
Asian	6101 (13%)	2969 (13%)	3132 (13%)
Other	8449 (18%)	4113 (18%)	4336 (18%)
Spending money			
$0	7297 (16%)	3866 (17%)	3431 (14%)
$1–20 (ref.)	11,156 (24%)	5257 (23%)	5799 (24%)
$21–100	11,964 (25%)	5339 (23%)	66624 (27%)
>$100	10,057 (21%)	5363 (23%)	4694 (19%)
I don’t know	6816 (14%)	3070 (14%)	3746 (16%)
Self-rated MH			
Poor	11,162 (24%)	3806 (17%)	7356 (30%)
Good (ref.)	36,128 (76%)	19,189 (83%)	16,939 (70%)
Emotion regulation Mean (SD)	14.257 (4.767)	13.311 (4.336)	15.152 (4.980)
Flourishing Mean (SD)	31.760 (5.684)	32.148 (5.627)	31.393 (5.713)
Family support			
Low	10,317 (22%)	4416 (19%)	5901 (24%)
Neutral/ambivalent	9614 (20%)	4594 (20%)	5020 (21%)
High (ref.)	27,359 (58%)	13,985 (61%)	13,374 (55%)
Peer Support			
Low	4419 (9%)	2238 (10%)	2181 (9%)
Neutral/ambivalent	7142 (15%)	3668 (16%)	3474 (14%)
High (ref.)	35,729 (76%)	17,089 (74%)	18,640 (77%)
School connectedness Mean (SD)	18.219 (0.641)	18.430 (3.430)	18.019 (3.237)
Bullying			
Not bullied (ref.)	40,089 (85%)	19,690 (86%)	20,399 (84%)
Bullied	7201 (15%)	3305 (14%)	3896 (16%)
Help-seeking			
Not reluctant (ref.)	20,091 (42%)	11,700 (51%)	8391 (35%)
Reluctant	27,199 (58%)	11,295 (49%)	15,904 (65%)

MH = Mental Health.

**Table 2:** School characteristics of the schools participating in Year 6 and 7 (2017–2018, 2018–2019) of the COMPASS Study in Canada (N = 116).

Variable	Total N (%)
Enrolment	
0–500	58 (50%)
500–1000 (ref.)	51 (44%)
1000–1500	7 (6%)
Urbanicity	
Rural/small urban	65 (56%)
Medium/large urban(ref.)	51 (44%)
School area median household income	
$25,000–50,000	14 (12%)
$50,000–75,000 (ref.)	69 (60%)
$75,000–100,000	28 (24%)
>$100,000	5 (4%)
Past prevalence of poor MH Mean (SD)	22.32 (8.16)
MH as a school priority	
Low	19 (16%)
High (ref.)	96 (84%)
MH professionals	
None	28 (24%)
Medium	73 (63%)
High (ref.)	15 (13%)
MH services	
Low	80 (69%)
High (ref.)	36 (31%)

MH = Mental Health.

**Table 3:** Adjusted odds ratio estimates for endorsing reluctance towards help-seeking using generalized equation estimation models.

Variable	Model I	Model II	Model III	Model IV
Intercept	1.31 (1.24, 1.38) ***	0.70 (0.60, 0.82)	3.21 (2.20, 4.69)	3.34 (2.27, 4.90) ***
Enrolment				
0–500		1.00 (0.93, 1.08)	0.96 (0.89, 1.03)	0.96 (0.89, 1.03)
500–1000 (ref.)		-	-	-
1000–1500		1.05 (0.96, 1.15)	0.99 (0.88, 1.12)	0.99 (0.88, 1.12)
Urbanicity				
Rural/small urban		0.87 (0.81, 0.94) **	0.85 (0.79, 0.93) **	0.86 (0.79, 0.93) **
Medium/large urban (ref.)		-	-	-
School area median household income				
$25,000–50,000		1.13 (0.99, 1.29)	1.16 (1.03, 1.30)	1.16 (1.03, 1.30)
$50,000–75,000 (ref.)		-	-	-
$75,000–100,000		1.03 (0.95, 1.12)	0.99 (0.91, 1.09)	1.00 (0.91, 1.09)
>$100,000		1.35 (1.19, 1.53) ***	1.20 (1.01, 1.43) *	1.20 (1.00, 1.43)
Past prevalence of poor MH		1.03 (1.02, 1.04) ***	1.01 (1.00, 1.01)	1.00 (1.00, 1.01)
MH as a school priority				
Low		0.96 (0.88, 1.06)	0.96 (0.86, 1.06)	0.96 (0.86, 1.06)
High (ref.)		-	-	-
MH professionals				
Low		0.99 (0.89, 1.10)	1.02 (0.90, 1.16)	0.98 (0.85, 1.13)
Medium		0.94 (0.87, 1.03)	1.03 (0.92, 1.15)	1.01 (0.89, 1.14)
High (ref.)		-	-	-
MH services				
Low		1.03 (1.02, 1.04)	0.97 (0.90, 1.06)	1.04 (0.88, 1.05)
High (ref.)		-	-	-
Sex				
Males (ref.)		-		-
Females			1.70 (1.63, 1.78) ***	1.70 (1.63, 1.78) ***
Grade				
9 (ref.)			-	-
10			1.03 (0.97, 1.09)	1.03 (0.97, 1.09)
11			1.09 (1.03, 1.15) **	1.09 (1.03, 1.15) **
12			1.04 (0.97, 1.12)	1.04 (0.97, 1.12)
Race/ethnicity				
White (ref.)			-	-
Black			0.87 (0.79, 0.97) **	0.86 (0.77, 0.97) **
Asian			0.86 (0.77, 0.97) **	0.87 (0.79, 0.97) **
Other			0.93 (0.88, 0.99) **	0.93 (0.88, 0.99)
Spending money				
$0			1.00 (0.93, 1.08)	1.01 (0.94, 1.08)
$1–20 (ref.)			-	-
$21–100			0.97 (0.91, 1.02)	0.95 (0.90, 1.00)
>$100			0.88 (0.91, 1.03)	0.97 (0.91, 1.03)
I don’t know			0.88 (0.88, 0.98) *	0.93 (0.88, 0.98)
Self-rated MH				
Poor			1.76 (1.65, 1.87) ***	1.75 (1.65, 1.86) ***
Good (ref.)			-	-
Emotion regulation			1.08 (1.07, 1.09) ***	1.08 (1.07, 1.09) ***
Flourishing			0.96 (0.96, 0.97) ***	0.96 (0.96, 0.97) ***
Family support				
Low			2.31 (2.16, 2.47) ***	1.94 (1.65, 2.29) ***
Neutral/ambivalent			1.74 (1.65, 1.84) ***	1.66 (1.41, 1.95) ***
High (ref.)			-	-
Peer Support				
Low			1.20 (1.10, 1.30) ***	1.31 (1.07, 1.60) **
Neutral/ambivalent			1.21 (1.13, 1.31) ***	1.09 (0.92, 1.28)
High (ref.)			-	-
School connectedness			0.93 (0.92, 0.93) ***	0.93 (0.92, 0.93) ***
Bullying				
Not bullied (ref.)			-	-
Bullied			1.17 (1.26, 1.26) ***	1.17 (1.09, 1.26) ***
Peer support * MH services				
Low, low				0.84 (0.68, 1.05)
Moderate, low				1.13 (0.99, 1.29)
Peer support * MH professionals				
Low, low				1.03 (0.79, 1.33)
Low, medium				1.03 (0.82, 1.28)
Moderate, low				1.05 (0.85, 1.30)
Moderate, medium				1.02 (0.85, 1.22)
Family support * MH services				
Low, low				1.13 (0.99, 1.29)
Moderate, low				0.99 (0.98, 1.11)
Family support * MH professionals				
Low, low				1.18 (0.97, 1.43)
Low, medium				1.09 (0.91, 1.31)
Moderate, low				1.07 (0.90, 1.27)
Moderate, medium				1.05 (0.90, 1.22)

Model I: No explanatory variables entered = help-seeking variable. Model II: School variables = help-seeking variable. Model III: School + student variables = help-seeking variable. Model IV: School + student + interaction variables = help-seeking variable. Model III and IV adjusts for Province. MH = Mental Health. Emotion regulation: higher scores represent greater socio-emotional skills. Flourishing: higher scores represent higher psychological well-being (flourishing), lower scores represent poorer psychological well-being (languishing). For interaction variables, “Moderate” = Neutral/Ambivalent. * = *p* < 0.05, ** = *p* < 0.01, *** = *p* < 0.001.

## Appendix A

**Mental Health Help-Seeking Attitudes**

If you had concerns regarding your mental health, are there any reasons why you would not talk to an adult at school (e.g., a school social worker, child and youth worker, counsellor, psychologist, nurse, teacher, or other staff person)? (Mark all that apply).

- I would have no problems talking to an adult at school about my mental health
- Worried about what others would think of me (e.g., I’d be too embarrassed)
- Lack of trust in these people—word would get out’
- Prefer to handle problems myself
- Do not think these people would be able to help
- Would not know who to approach
- There is no one I feel comfortable talking to

**School-Level Variables**

**Mental Health as a School Priority**

Please rank these school/health-related issues in terms of importance to your school: (Rank items from 1 to 10 where 1 = highest priority and 10 = lowest priority)

- Tobacco use
- Alcohol and other drug use
- Healthy eating
- Physical activity
- Bullying/violence
- Mental health
- Sexual health
- Sun safety/tanning beds
- Obesity/overweight/healthy weight
- Sedentary behaviours/screen time

**Availability of Mental Health Professionals**

Please indicate the availability of the following mental health professionals at your school (Select all availability options that apply)

(Response options: on-call, on-site full time, regularly scheduled __ hours/month)

- Child and Youth Worker
- Counsellor
- Social Worker
- Psychologist
- Mental Health Nurse
- Other (please list):

**Availability of Mental Health Services**

Are any of the following mental health services available on-site at your school? (*Check all that apply)*

- Assessment for emotional or behavioural problems (including behavioural observation, psychosocial assessment and observation checklists)
- Diagnostic assessment (comprehensive psychological evaluation)
- Behavioural management consultation with teachers, students, or families
- Case management, including monitoring and coordination of services
- Referral to specialized programs or services for emotional or behavioural problems or disorders
- Crisis intervention (e.g., response to traumatic events, including disasters, serious injury/death of a member of the school community)
- Individual counselling/therapy
- Group counselling/therapy
- Substance abuse counselling
- Family support services in school setting (e.g., child/family advocacy, counselling)

**Student-Level Variables**

**Sex**

Are you female or male?

- Female
- Male

**Grade**

What grade are you in?

- Grade 9
- Grade 10
- Grade 11
- Grade 12

**Race/Ethnicity**

How would you describe yourself? *(Mark all that apply)*

- White
- Black
- Asian
- Aboriginal (First Nations, Métis, Inuit)
- Latin American/Hispanic
- Other

**Spending Money**

About how much money do you usually get each week to spend on yourself or to save?

*(Remember to include all money from allowances and jobs like baby-sitting, delivering papers, etc.)*

- Zero
- $1 to $3
- %6 to $10
- $11 to $20
- $21 to $40
- $41 to $100
- More than $100
- I do not know how much money I get each week

**Self-Rated Mental Health**

In general, how would you rate your mental health?

- Excellent
- Very good
- Good
- Fair
- Poor

**Emotion Regulation**

Please indicate how often the following statements apply to you:

(Response options: almost never, sometimes, about half the time, most of the time, almost always)

(a) I have difficult making sense out of my feelings
(b) I pay attention to how I feel
(c) When I’m upset, I have difficulty concentrating
(d) When I’m upset, I believe there is nothing I can do to make myself feel better
(e) When I’m upset, I lose control over my behaviour
(f) When I’m upset, I feel ashamed for feeling that way

**Flourishing**

How much do you agree or disagree with the following statements?

(Response options: strongly agree, agree, neither agree nor disagree, disagree, strongly disagree)

(a) I lead a purposeful and meaningful life
(b) My social relationships are supportive and rewarding
(c) I am engaged and interested in my daily activities
(d) I actively contribute to the happiness and well-being of others
(e) I am competent and capable in the activities that are important to me
(f) I am a good person and live a good life
(g) I am optimistic about my future
(h) People respect me
(i) I generally recover from setbacks quickly

**Family Support**

How much do you agree or disagree with the following statements?

I can talk about my problems with my family

- Strongly agree
- Agree
- Neither agree nor disagree
- Strongly disagree

**Peer Support**

How much do you agree or disagree with the following statements?

I can talk about my problems with my friends

- Strongly agree
- Agree
- Neither agree nor disagree
- Strongly disagree

**School Connectedness**

How strongly do you agree or disagree with each of the following statements?

(Response options: strongly agree, agree, disagree, strongly disagree)

(a) I feel close to people at my school
(b) I feel I am part of my school
(c) I am happy to be at my school
(d) I feel the teachers at my school treat me fairly
(e) I feel safe in my school
(f) Getting good grades is important to me
